# Online Sobriety Communities for Women's Problematic Alcohol Use: A Mini Review of Existing Qualitative and Quantitative Research

**DOI:** 10.3389/fgwh.2021.773921

**Published:** 2021-12-09

**Authors:** Claire Davey

**Affiliations:** Faculty of Arts and Humanities, Canterbury Christ Church University, Canterbury, United Kingdom

**Keywords:** online sobriety communities, women's recovery, temporary abstinence campaigns, alcohol online support groups, women in sobriety

## Abstract

The increase in women's drinking is one of the most prominent trends in alcohol consumption in the UK in recent history, possibly exacerbated by COVID-19 lockdown measures. Higher rates of drinking are associated with substantial economic, health, and social costs. However, women are less likely to seek treatment for Alcohol Use Disorder (AUD) than men and have less successful treatment outcomes from traditional treatment paths, such as 12-step programs and in-patient care. Female heavy drinkers may also experience particular forms of gendered stigma that affect their experiences of addiction and recovery and their desire or ability to access these more “traditional” services. This review provides an overview of existing qualitative and quantitative research regarding online sobriety communities that are predominantly utilised by women, such as non-12-step alcohol online support groups (AOSGs) and temporary abstinence initiatives (TAIs). This is a small—but expanding—body of literature emerging as “sober curiosity” and “mindful drinking” are trending in Western contexts such as the UK, particularly amongst young women who do not identify with traditional, binary recovery language such as “alcoholic” and “addict.” This review highlights the gaps in research and concludes that further research regarding these new treatment pathways, and women's experiences when utilising them, must be conducted to provide more evidence-based options for women who want to address problematic drinking. Public health bodies could also learn more effective strategies from these innovative solutions to reduce alcohol consumption generally.

## Introduction

Despite the substantial economic, health, and social burden of alcohol on the UK economy (1), the affordability and availability of alcohol in England and Wales has significantly increased during the post-war years (2, 3). Consumption reached a peak in 2004 at an average of 11.6 l consumed per person across the annual period (4). The most significant driver of this trend was the substantial rise in women's drinking (5) and yet women's alcohol consumption remains a considerably under-explored area of research compared to that of men's. The global COVID-19 pandemic (6) and resulting UK lockdown restrictions more recently impacted drinking behaviours for both men and women. It is estimated that around a quarter of the population drank less than usual, and around a quarter drank more (7), though young women's drinking was identified to be disproportionately exacerbated (8). This is likely due to the burdensome impacts of the pandemic—such as care obligations and employment precarity—that were additionally disproportionately experienced by women (9–11).

A substantial body of research shows that women are far less likely to seek help for problematic drinking from traditional, evidence-based treatment programs (12) (which are also the most frequently researched programs), including disease model or 12-step approaches such as Alcoholics Anonymous (13, 14), CBT models such as SMART recovery (15), and those based on Recovery Capital (16). There are a number of driving factors for this lack of engagement. From a practical perspective, women may find it harder to attend treatment outside of the home, particularly residential programs, due to family and work commitments (12). Further, women face more barriers to access and experience disproportionate shame when they do access treatment due to their perceived failure to live up to society's expectations of womanhood (12, 17). Problematic drinking by women contradicts traditional notions of respectable femininities (passive, quiet, nurturing) and women's gender roles (mother, wife, carer) (18–20). Thus, a woman seeking help for addiction is more likely to be viewed as a “deviant…moral failure” than her male peer [(13), p. 146–7].

The under-representation of women within traditional treatment programs suggests that there is a failure to recognise women's gendered experiences of alcohol and specific needs in recovery (21, 22). Women-only treatment provision may lower the barriers to access for women, and encourage more to seek help, but the provision of women-only treatment does not predicate significant differences in treatment outcomes unless paired with treatment which caters to women's gendered needs, as outlined above (16, 23–26). For instance, it has been found that women prefer more comprehensive ideals of success that are not only based on abstinence (13), but also allow for moderation (27). It has also been found that women prefer a more positive, self-reliant approach rooted in ideas of self-development (14, 17).

With this in mind, it is perhaps unsurprising that the internet has provided an expansion of recovery modalities which have sought to distance themselves from more traditional recovery programs. These groups and communities move away from the use of binary language such as “alcoholic” and “addict” in the ways in which they depict and promote “alcohol-free” living, with a view to reducing the stigma and shame that is attached to alcohol refusal (particularly for women). The last 10 years have seen international growth in the number and utilisation of web-based non-12-step alcohol online support groups (AOSGs) such as Soberistas, and later, social media-based sobriety communities such as Club Soda UK, Soberful, LoveSober and Sober Girl Society (to name but a few). These predominantly offer peer support, information provision and recovery coaching services to those who want to renegotiate their relationship with alcohol, irrespective of where they are on the continuum of alcohol consumption. They are typically based on a variety of “for profit” business models with some degree of free access or content.

During the same time period, there has been a rise in global popular engagement with temporary abstinence initiatives (TAIs). Australian charity Hello Sunday Morning (28) was the first to gain popularity with their user-driven blogging site in 2010, followed promptly by the launch of Alcohol Change UK's Dry January (29) campaign in 2013. Keen to mirror the UK's success, Kék Pont Alapítvány (Blue Point Foundation), a substance use NGO, started Dry November (30) in Hungary in 2015. Other campaigns have subsequently been launched across these territories, such as Macmillan's Sober October (31) in the UK, and Febfast (32) and Dry July (33) in Australia. These temporary abstinence initiatives are all organised by third sector organisations and challenge participants to complete a month of abstinence from alcohol in a bid to reduce alcohol harms or to raise money for charitable causes. They typically disseminate campaign content via email and their websites, and some create peer to peer online support communities. In creating a shared recovery experience, mediated through the internet, these TAIs can be considered as an extension of the online sobriety communities outlined above.

**Figure 1:**
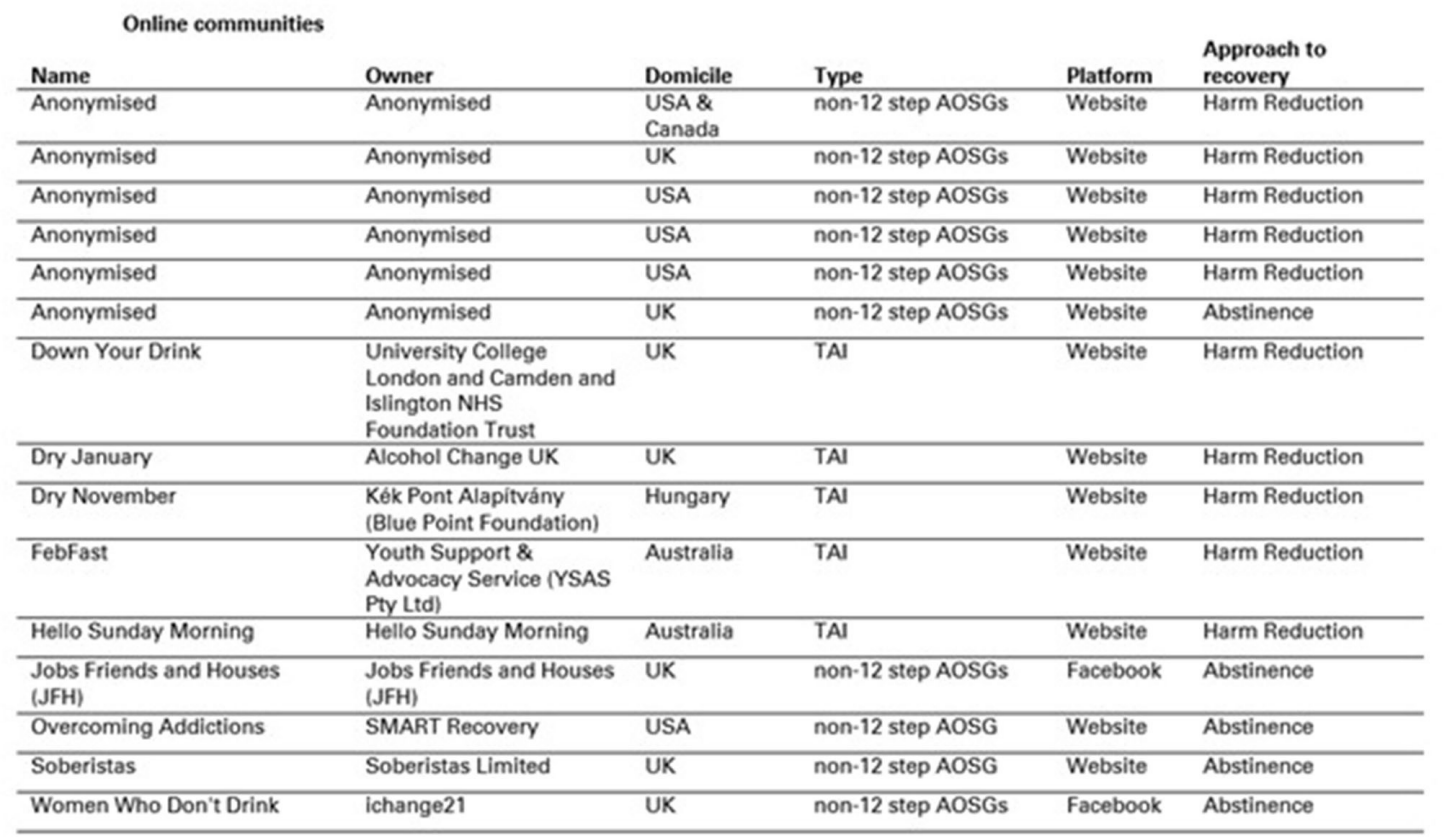
Online communities.

The twenty-first century has seen the emergence of studies which consider the online support offerings available for different health concerns (34–37), including problem drinking. This article provides a focused literature review of existing qualitative and quantitative research regarding the online sobriety communities (non-12-step AOSGs and TAIs) outlined in Figure 1, particularly pertaining to the demographics of their participants, why and how participants use them, and their efficacy in changing drinking behaviours. In doing so, I will highlight some limitations within the existing research and some of the gaps that remain. Final conclusions will provide some suggestions as to how the findings regarding non-12-step AOSGs and TAIs can inform subsequent research regarding the popular, social-media-based sobriety communities, and identify the value such research has beyond the academic community.

All papers regarding AOSGs and TAIs reviewed within this article were written in English and published between 2000 and 2020. The review was conducted via snowballing methods, including bibliographic searches and citation tracking, which are often employed for niche areas of research where publications are limited in volume. This method mitigates dependence upon key terms or content within specific technology platforms and avoids the subjectivity of algorithms that can perpetuate the under-representation and under-citation of some authors' research—particularly women and those of colour. However, there are limitations in this method, including human subjectivity regarding selection criterion, and a reduced focus on the meta-data.

## Women's Participation in Online Sobriety Communities

Existing research suggests that participants within non-12-step AOSGs and TAIs are disproportionately female compared to the demographics of those presenting for publicly-funded treatment. Graham et al.'s analysis of the 2015 UK Life in Recovery survey results found that there was a significant association between gender and the use of online recovery groups (38). More nuanced studies support this: Sinclair et al., found that 94% of respondents to their study of Soberistas were female, “overwhelmingly in employment with post-graduate qualifications” [(39), p. 223–4]—a demographic reflected in Sanger et al.'s study of multiple non-12-step AOSGs (40). Furthermore, research regarding Dry January (34) and Hello Sunday Morning (41, 42) shows that women are also at the forefront of trying to renegotiate their relationship with alcohol via abstinence challenges. Those who attempted to have a dry January in 2019 were more likely to be women, of higher socio-economic status and more health conscious (43). However, it must be acknowledged from this evidence that non-12-step AOSGs and TAIs do not attract a diverse demographic in terms of class and race. More research must be done to determine why these initiatives appeal to specifically white females of higher socioeconomic capital, and thus better understand who they unconsciously exclude and why.

In her review of Australian TAIs, Robert questions these initiatives' abilities to access “those who have a problem with alcohol” or “irresponsible drinkers” [(44), p. 654]. She suggests that themes of self-care, self-improvement and philanthropy appeal to an already-“responsibilised” demographic of neoliberal subjects ((44), p. 647). The predominantly late-twenties-to-middle-aged, middle-class, female face of a TAI participant does not align with the data of those who access Australian, government-funded alcohol interventions and therefore the possibility of this demographic experiencing problematic drinking is dismissed by Robert. Nor, perhaps, is this demographic visible in the public displays of “binge drinking” culture that are so often the hyperbolised target of class-driven media and policy-maker scrutiny (45, 46). Yet research into both Hello Sunday Morning (41, 42) and Dry January (43) suggests that participants are often risky or high-risk consumers of alcohol prior to participation—data which is also reflected in Sinclair et al.'s study of non-12-step AOSG Soberistas (39). Thus, another conclusion could be that the demographics of Alcohol Use Disorders are changing, and with the increasing plethora of treatment paths available it could be the case that more women are reaching out for help via a modality that appeals to them. Furthermore, online communities may reduce or remove the aforementioned gendered barriers to treatment, as the next section will go on to explore.

## Meeting Women's Needs in Recovery

Studies of non-12-step AOSGs and TAI communities suggest a multitude of reasons why people (particularly women) are increasingly utilising internet platforms, including; flexibility and access (40, 47, 48), accountability (48), stigma reduction through anonymity (39, 48, 49), specialist information (39, 50), support (39, 42, 51, 52), empowerment (53), and the value of sharing personal storeys in the written form (39, 49, 50, 54). Many of these advantages align with the gendered needs of women in recovery outlined within the introduction. Online modalities can be juggled with work or care responsibilities, and the possibilities of stigmatisation and shame are mitigated if platform use is anonymous and/or mediated through the written word.

Sanger et al. found that the most important benefit of non-12-step AOSGs cited by partiwas the ability to meet “someone like me,” who shared the same experiences and did not adhere to popular stereotypical connotations of an “alcoholic” [(40), p. 2]. While Soberistas endorses a path of abstinence, it more closely aligned with popular themes of an “alcohol-free” lifestyle and self-improvement (19, 48), similar to TAIs Hello Sunday Morning (41) and Dry January (55). This ability to individually self-define, reframe and re-work traditional terms (19, 20, 40, 41, 56) is valued by participants, and reduces the stigma associated with the false binary of “addict” or “alcoholic” vs. “normal” drinker (57). These groups provided “a sense of normality for those who did not feel they belonged in the world of AA” [(40), p. 3]. Additionally, participants within Khadjesari et al.'s study almost unanimously suggested that they perceived a lack of treatment options for those who wanted to moderate as opposed to abstain, and that the goal of abstinence was a barrier to seeking help (49). This aligns with previously cited research which suggests that women prefer more comprehensive ideals of success that are not only based upon abstinence (13).

There is very limited research that explores the gendered nature of communications within online sobriety communities. Carah et al. (41) found that female participants of Hello Sunday Morning were more likely to pursue body and alcohol-related goals, whilst Klaw et al. (50) found no gender differences within communication patterns of their sample. Other studies of content within non-12-step AOSGs did not pursue this avenue of investigation, and Yeomans's analysis of 2017's Dry January media content and Facebook group posts/comments also did not consider gender in its qualitative review (55). The ways in which women communicate and perform their identity in sobriety, within online communities, could be relevant to the design of treatment, and its outcomes and therefore should be considered within future research.

## Efficacy of Online Sobriety Communities

It is difficult to verify the extent to which participants engage with the online sobriety communities. Quantitative data provided to Sinclair et al. (39) in their mixed-methods study of web-based Soberistas suggests that the majority (2000) of its 3,828 active users were only “browsers”—most reported “time spent lurking and passively consuming…without actively contributing to it” [(48), p. 7]. Within Hello Sunday Morning's forum, Carah et al. (41) found that 59% of content posted was contributed by only 16% of its users. Indeed, size of the community can also be a factor in this; if it is too large, participants struggle to keep up with the flow of content and can become disengaged (40), yet being active in the community, feeling central to the group, and being endorsed for contributions, have been found to support the recovery process (58). Furthermore, this dynamic can also be seen within Dry January campaigns whereby vast numbers (6.5 million) allegedly attempt the month of abstinence without signing up to the official campaign (29), and those who do register [100,000 in 2020 (59)] may not fully engage with the content provided. Ultimately those who *did* read all supportive email campaign content were more likely to have successfully completed the challenge (60). Thus, more research could be done to determine the links between participant engagement within online sobriety communities and treatment outcomes in order to drive improvement.

The efficacy of online sobriety communities in reducing alcohol consumption has been the subject of some quantitative and mixed-methods research. A range of findings across non-12-step AOSG interventions show that the majority of participants reduced their alcohol consumption as a result of engaging with these initiatives (15, 39, 47), or were more likely to continue with treatment (58). For instance, a small sample from SMART recovery found that their online intervention, Overcoming Addictions, was just as effective in helping people recover from problem drinking as the traditional, in-person approach (15). Furthermore, 55% of those who took part in Sinclair et al.'s online survey of Soberistas became alcohol-free or reduced consumption post joining (39). It has also been found that completing a month of abstinence, via Dry January, led to significantly higher levels of self-reported well-being and general self-efficacy amongst participants (60). This was subsequently bolstered by findings which suggest that the health benefits gained from completing Dry January are maintained by participants at a 6-month follow-up, including reduced levels of alcohol consumption than prior to the month of abstinence (43). However, limitations in these findings regarding efficacy include relatively small sample sizes, participant self-selection, and the drop-off in sample sizes at follow-ups. Furthermore, while the publicly-stated community goals of either abstinence or harm reduction are known (see Figure 1), the extent to which their participants aspired to these goals cannot be determined. More work could be done to verify these emerging findings.

The multitude of offerings within online sobriety communities and porous community boundaries suggests that in isolation they do not provide a “one stop shop” solution to recovery—participants leave or join groups at different stages in their recovery (40, 48). For example, Sanger et al., found that some non-12-step AOSG participants used the communities in conjunction with in-person AA meetings (40). However, the existing research does not explore in detail how participants engage with multiple tools and discourses in order to develop a holistic recovery strategy and reach their personal goals regarding alcohol (non) consumption. Furthermore, the quantitative research regarding Dry January (43, 60), and other TAIs (42, 51) typically portrays individuals' sobriety journeys to start and end within a calendar month—or 6 months if follow-up is included (43)—yet their activity and involvement within the broader sobriety community may have greater longevity. Existing studies consider participants' engagement with TAIs in isolation, which provides a one-dimensional view of their relationship with alcohol. A greater dialogue with participants, using qualitative methods, is required in order to establish whether they utilise TAIs in tandem with other recovery support, particularly other online sobriety communities. Additionally, it would be helpful to understand whether TAIs make participants “sober-curious” (61) and thus act as a gateway into other online sobriety communities.

## Conclusion

This review is the first to map the existing literature regarding online sobriety communities: non-12-step alcohol online support groups and temporary abstinence initiatives. In doing so, it has compiled the limited evidence to suggest that these alternative treatment paths can be effective in helping individuals to overcome problematic drinking. It also suggests that they reduce the barriers to women's access to treatment through flexibility, anonymity, and like-minded support via the sharing of personal storeys in the written form. This review also highlighted the value of non-binary language regarding alcohol consumption and treatment goals in order to create an inclusive space for women. However, there are still opportunities for online sobriety communities to broaden their demographic in terms of gender, race, class, and sexuality. Subsequent research could instead explore smaller communities that are tailored to the needs or cultural requirements of individuals within these different demographics, such as Club Soda's Queers Without Beers and Sober Black Girls Club.

The limitations of the existing literature have additionally been highlighted, including data sampling considerations. There are also the constraints inherent within quantitative methods when seeking to understand experiences within communities that use the written form. Contained within this review are a range of qualitative, quantitative and mixed-method studies yet none employed ethnographic methods. Future research would benefit from the utilisation of sociological ethnographic methods (62, 63) to gain a deeper understanding of women's experiences and identities—both within online sobriety communities and beyond. Furthermore, due to the gendered stigma and shame experienced by women in recovery, such research may benefit from an “intimate insider” (64) researcher who is able to illicit the trust of the users of the communities in order to further understand their participation and accurately represent their experiences.

It was identified that there is currently no published research regarding the current, popular social media-based sobriety communities such as Club Soda UK, Soberful, LoveSober and Sober Girl Society. While Nicholls' “Sobriety Storeys” project recruited women from a social media-based AOSG, it did not explore the relationship between social media and women's identity construction within the recovery community (19, 20). This would be of value. It is known that social media sites are spaces used by women to enhance drinking practises—to heighten fun, popularity and bonding, and facilitate displays of heteronormative, hyper-sexualities and femininities (65–67), yet future research should seek to understand the role of social media in “doing” sobriety and non-drinking practises.

This review offers possibilities of alternative paths to recovery. It has shown that participation within online treatment paths does not have to be an either/or binary decision (68) but can supplement the use of in-person or other more “traditional” modalities. It has presented findings which are of value to the online sobriety communities themselves, for both temporary and longer-term initiatives. These insights could help shape future strategies and encourage their engagement in future research projects. In addition, public health bodies could use this emerging evidence regarding online interventions to develop future policies and funding decisions that seek to reduce alcohol related harms across the population. Lastly, practitioners could consider directing patients to some of these initiatives if they are not eligible for publicly funded treatment.

## Author Contributions

CD was responsible for the conception, structure, analysis, and writing within this paper.

## Funding

CD was supported by a University Research Scholarship at Canterbury Christ Church University.

## Conflict of Interest

The author declares that the research was conducted in the absence of any commercial or financial relationships that could be construed as a potential conflict of interest.

## Publisher's Note

All claims expressed in this article are solely those of the authors and do not necessarily represent those of their affiliated organizations, or those of the publisher, the editors and the reviewers. Any product that may be evaluated in this article, or claim that may be made by its manufacturer, is not guaranteed or endorsed by the publisher.
